# Improved assessment of aortic hemodynamics by *k-t* accelerated dual-*venc* 4D flow MRI in pediatric patients

**DOI:** 10.1186/1532-429X-18-S1-O96

**Published:** 2016-01-27

**Authors:** Susanne Schnell, Michael J Rose, Can Wu, Julio Garcia, Joshua D Robinson, Michael Markl, Cynthia K Rigsby

**Affiliations:** 1 grid.465264.7Dept. of Radiology, Northwestern University, Feinberg Medical School, Chicago, IL USA; 2 grid.413808.60000000403882248Pediatric Radiology, Lurie Children's Hospital of Chicago, Chicago, IL USA; 3 grid.16753.360000000122993507McCormick School of Engineering, Northwestern University, Evanston, IL USA; 4 grid.413808.60000000403882248Pediatrics-Cardiology, Lurie Children's Hospital of Chicago, Chicago, IL USA

## Background

Bicuspid aortic valve (BAV) is often associated with aortic stenosis, resulting in high velocity blood flow in the ascending aorta, with slow flow areas in the dilated aorta or adjacent vessels. Similarly, Marfan syndrome patients develop aortic root dilatation and regurgitation with both high and low velocity blood flow. 4D flow MRI is typically acquired with a velocity encoding (*venc*) gradient adjusted to the estimated peak velocity. As the velocity noise is directly related to the velocity sensitivity (σ*v*~*venc*), a high *venc* can substantially limit the assessment of low flow velocities (*v* < < *venc*). We therefore developed a fully integrated low- and high-*venc* PC-MRI in a single measurement (dual-*venc*) to avoid aliasing and improve velocity-to-noise ratio (VNR).

## Methods

In addition to standard clinical contrast enhanced cardiac MRI, *k-t* GRAPPA accelerated dual-*venc* 4D flow MRI was acquired with full volumetric coverage of the ascending (AAo) and descending (DAo) aorta in 4 Marfan syndrome patients (age = 16.3 ± 1.9 yrs, 1 female, low-*venc* = 81 cm/s, high-*venc* = 200 cm/s) and 1 BAV patient (age = 20 yrs, male, low-*venc* = 125 cm/s, high-*venc* = 350 cm/s) on a 1.5T Siemens MAGNETOM Aera MRI scanner with the following imaging parameters: R = 5, TE/TR = 2.93 ± 0.08/5.46 ± 0.08ms, voxel size = 1.9 × 1.9 × 2.0 mm^3^. Background correction was performed separately of the low- and high-*venc* phase images. High-*venc* data were used for complete anti-aliasing of the low-*venc* data while maintaining the favorable VNR of the low-*venc* data. A pseudo-complex MRA was derived from the 4D flow data and used to segment the angiogram using commercial software (MIMICS, Materialize). Velocity noise was estimated in static tissue using region of interest analysis. Velocity maximum intensity projection maps (MIPs) were calculated and used to determine peak velocities in the overall aorta and in the three sub-segments AAo, Arch and DAo. In addition, blood flow was visualized using streamlines within the segmented vessels during peak systole.

## Results

Velocity noise was significantly improved in the dual-*venc* 4D flow data by 230% ± 20% compared to the high-*venc* velocity scan for the four Marfan patients, which corresponded with the high-*venc*/low-*venc* ratio of 2.5. In the BAV patient, velocity noise was improved by 276% in the dual-*venc* compared to the high-*venc* scan (high-*venc*/low-*venc* ratio=2.4). Peak velocity showed significant correlation between high- and dual-*venc* results in the AAo (R^2^ = 0.85, p = 0.04) and Arch (R^2^ = 0.95, p = 0.006) using velocity MIP analysis. Streamline comparison showed substantially improved qualitative visualization of aortic blood flow patterns and more coherent streamlines especially in slow flow areas and no aliasing artifacts in areas that were aliased in the low-*venc* images.

## Conclusions

This feasibility study demonstrates the potential of *k-t* accelerated dual-*venc* 4D flow MRI to improve the assessment of aortic hemodynamics with large dynamic range while maintaining the scan time of standard 4D flow MRI.

**Figure 1:**
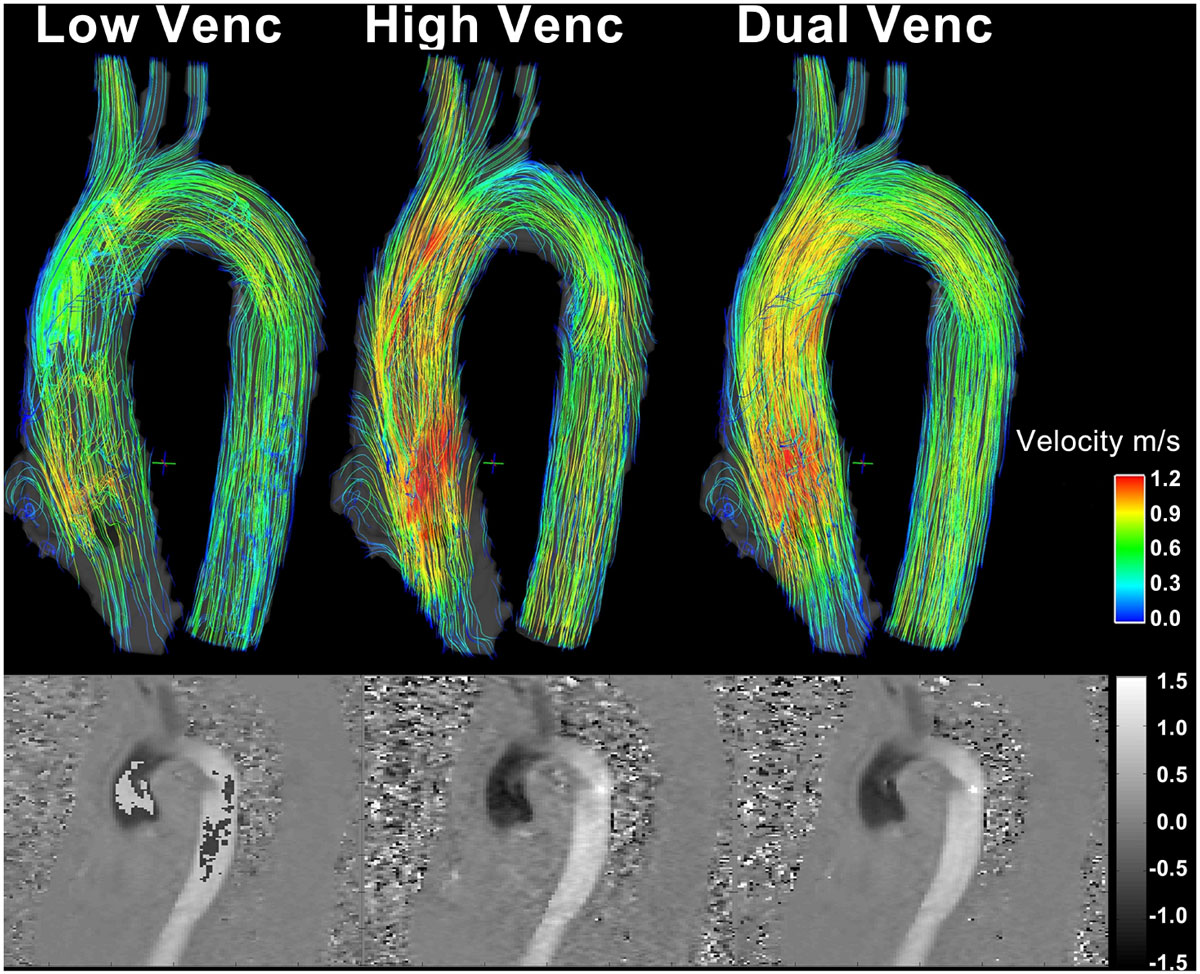
Figure 1

